# Non-Invasive Measurement of Skin Microvascular Response during Pharmacological and Physiological Provocations

**DOI:** 10.1371/journal.pone.0133760

**Published:** 2015-08-13

**Authors:** Fredrik Iredahl, Andreas Löfberg, Folke Sjöberg, Simon Farnebo, Erik Tesselaar

**Affiliations:** 1 Department of Clinical and Experimental Medicine, Linköping University, Linköping, Sweden; 2 Department of Plastic Surgery, Hand Surgery, and Burns, Linköping University, Linköping, Sweden; 3 Department of Radiation Physics, Linköping University, Linköping, Sweden; Université Claude Bernard Lyon 1, FRANCE

## Abstract

**Introduction:**

Microvascular changes in the skin due to pharmacological and physiological provocations can be used as a marker for vascular function. While laser Doppler flowmetry (LDF) has been used extensively for measurement of skin microvascular responses, Laser Speckle Contrast Imaging (LSCI) and Tissue Viability Imaging (TiVi) are novel imaging techniques. TiVi measures red blood cell concentration, while LDF and LSCI measure perfusion. Therefore, the aim of this study was to compare responses to provocations in the skin using these different techniques.

**Method:**

Changes in skin microcirculation were measured in healthy subjects during (1) iontophoresis of sodium nitroprusside (SNP) and noradrenaline (NA), (2) local heating and (3) post-occlusive reactive hyperemia (PORH) using LDF, LSCI and TiVi.

**Results:**

Iontophoresis of SNP increased perfusion (LSCI: baseline 40.9±6.2 PU; 10-min 100±25 PU; p<0.001) and RBC concentration (TiVi: baseline 119±18; 10-min 150±41 AU; p = 0.011). No change in perfusion (LSCI) was observed after iontophoresis of NA (baseline 38.0±4.4 PU; 10-min 38.9±5.0 PU; p = 0.64), while RBC concentration decreased (TiVi: baseline 59.6±11.8 AU; 10-min 54.4±13.3 AU; p = 0.021). Local heating increased perfusion (LDF: baseline 8.8±3.6 PU; max 112±55 PU; p<0.001, LSCI: baseline 50.8±8.0 PU; max 151±22 PU; p<0.001) and RBC concentration (TiVi: baseline 49.2±32.9 AU; max 99.3±28.3 AU; p<0.001). After 5 minutes of forearm occlusion with prior exsanguination, a decrease was seen in perfusion (LDF: p = 0.027; LSCI: p<0.001) and in RBC concentration (p = 0.045). Only LSCI showed a significant decrease in perfusion after 5 minutes of occlusion without prior exsanguination (p<0.001). Coefficients of variation were lower for LSCI and TiVi compared to LDF for most responses.

**Conclusion:**

LSCI is more sensitive than TiVi for measuring microvascular changes during SNP-induced vasodilatation and forearm occlusion. TiVi is more sensitive to noradrenaline-induced vasoconstriction. LSCI and TiVi show lower inter-subject variability than LDF. These findings are important to consider when choosing measurement techniques for studying skin microvascular responses.

## Introduction

Human microvascular function and specific physiological mechanisms of the microcirculation can be assessed in the skin by measuring vascular responses to various provocations. Transdermal iontophoresis can be used to stimulate pharmacological provocations in the skin, by delivering agonists or antagonists that specifically target receptors or pathways involved in microvascular regulation [1]. The technique has been used to deliver various agonists, including acetylcholine and sodium nitroprusside [2], insulin [3–5], noradrenaline and phenylephrine [6]. It has also been used to deliver receptor antagonists such as endothelin receptor antagonists [7], atropine [8], adrenoceptor antagonists [9] and the NOS pathway inhibitor L-NAME [3]. Local delivery of these active substances to the skin, possibly in combination with systemic pre-treatment [10], provides an elegant vascular pharmacological in vivo model [1, 11].

An alternative way to assess microcirculation in the skin is by physiological provocation. The most commonly used physiological provocations for assessment of endothelial function are arterial occlusion of the forearm (post-occlusive reactive hyperemia, PORH) [12–14] and local heating of the skin [15, 16].

Changes in microvascular reactivity in the skin have been shown to mimic systemic vascular function. For example, the response to iontophoresis of acetylcholine is impaired in patients with type-I diabetes [17] and in patients with increased risk of coronary heart disease [18, 19], while responses to PORH and local heating have both been found to be related to systemic endothelial function [20].

Traditionally, laser Doppler Flowmetry (LDF) has been the preferred technique for quantifying skin microvascular response. However in recent years new technologies, such as Laser Speckle Contrast Imaging (LSCI) [21, 22] and Tissue Viability Imaging (TiVi) [23], have been introduced. These camera-based techniques take advantage of both high spatial and high temporal resolutions. There are, however, important differences between the techniques and the measures they provide, which should be taken into account when measuring responses to vascular provocations in the skin (Table 1). Previous studies have focused on comparing technical aspects of the instruments [24], but how well the techniques are suited to specific types of vasoactive pharmacological and physiological provocations, including iontophoresis, local heating, tissue exsanguination, occlusion and PORH, has not been studied in detail.

**Table 1:** Technical characteristics of laser Doppler flowmetry (LDF), Laser Speckle Contrast Imaging (LSCI) and Tissue Viability Imaging (TiVi).

	LDF	LSCI	TiVi
**Measurement principle**	Doppler effect	Speckle contrast	Polarization spectroscopy
**Measurement parameter**	Perfusion	Perfusion	Red blood cell concentration
**Spatial resolution**	*	500 μm	50 μm
**Measurement area**	~ 1 mm^2^	15x15 cm^2^	40x40 cm^2^
**Temporal resolution**	33 Hz	Up to 44 images/s	Up to 25 images/s
**Measurement depth**	~ 0.5 mm	~ 0.5 mm	~ 0.5 mm

*LDF*: *laser Doppler flowmetry*, *LSCI*: *laser speckle contrast imaging*, *TiVi*: *Tissue Viability Imaging*, *fps*: *frames per second*.

* *since LDF measures perfusion in a single-point*, *the spatial resolution (distance between adjacent measurement points) is not defined*.

The purpose of this study was therefore to assess microvascular responses during pharmacological and physiological provocations to the skin of healthy subjects. TiVi, which measures the concentration of red blood cells (RBC), was compared to an established single-point technique (LDF), and an imaging technique (LSCI), which both measure perfusion. Separate experiments were performed to measure microvascular responses in the forearm skin during (1) iontophoresis of noradrenaline and sodium nitroprusside, (2) local heating and (3) post-occlusive reactive hyperemia following arterial occlusion with or without prior exsanguination (Table 2). We hypothesized that the differences between measurement properties of LDF, LSCI and TiVi affect their sensitivity to changes in microvascular responses during the vasoconstrictive and vasodilatory provocations.

**Table 2:** Overview of provocations and their underlying mechanisms. Six different skin sites on the forearm were used in three different experiments (Fig 1).

Provocation	Mechanism	Measurement technique	Site
Iontophoresis of SNP	Nitric oxide release, smooth muscle relaxation	LSCI, TiVi	1a
Iontophoresis of NA	Alpha-adrenergic stimulation, smooth muscle contraction	LSCI, TiVi	1b
Local heating	Biphasic: (I) activation of cutaneous sensory nerves (axon reflex); (II) nitric oxide release, smooth muscle relaxation.	LSCI, TiVi	2a
		LDF	2b
Exsanguination, occlusion, PORH	(I) Ischemia by exsanguination and total occlusion; (II) Recirculation of accumulated vasodilatory metabolites, smooth muscle relaxation.	LSCI, TiVi	3a
		LDF	3b

(1) Vasoactive drugs were delivered using single 20-μA current pulses with a duration of 10 min; (2) The skin was locally heated using a thermostatic laser Doppler probe or a custom-made transparent heating glass (Tesselaar et al., 2012); (3) Forearm occlusion followed by post-occlusive reactive hyperemia (PORH) was performed with and without prior exsanguination.

*NA*: *noradrenaline*, *SNP*: *sodium nitroprusside*, *LDF*: *laser Doppler flowmetry*, *LSCI*: *laser speckle contrast imaging*, *TiVi*: *Tissue Viability Imaging*, *PORH*: *post-occlusive reactive hyperemia*

## Methods

### Subjects

Twenty-six healthy, non-smoking subjects were recruited and gave their written informed consent. None of the subjects used regular medication, except for oral contraceptives, or had any known skin diseases. All were normotensive (defined as duplicate office measurement <140/<90). The subjects were asked to refrain from drinking coffee, tea, and alcohol, and from exercise on the day of the experiment. They did not eat or drink (except water) for 2 hours before the experiment. The subjects were seated comfortably in a semi-supine position during the measurements, which started after 20 minutes of acclimatization. All provocations and measurements were done on the volar forearm after the skin had been gently cleaned with chlorhexidine ethanol (5 mg/ml, Fresenius AB, Uppsala, Sweden). Blood pressure was measured before the start of measurement using an automatic sphygmomanometer (M6 Comfort, Omron Healthcare, Hoofddorp, The Netherlands). Lighting conditions in the room were kept constant by closing window blinds and turning off ceiling lights, and there was typically sufficient light to be able to read. All measurements were performed at a room temperature of 21 ± 1°C. The study was carried out according to the Declaration of Helsinki and was approved by the Regional Ethics Committee at Linköping University Hospital.

### Drugs

All drugs were obtained from the university hospital pharmacy. Sodium Nitroprusside (SNP, Nitropress 25mg/mL, Hospira Inc., Lake Forest, USA) was dissolved in sterile water to a final concentration of 10 mg/mL. Noradrenaline (NA) 1 mg/ml (APL AB, Umeå, Sweden) was used undiluted. The drugs were stored in the dark as recommended by the manufacturer.

### Equipment

#### Laser Doppler flowmetry

A laser Doppler perfusion monitor (Periflux 5000, Perimed AB, Järfälla, Sweden) with thermostatic laser Doppler probes (Probe 457, Perimed AB, Järfälla, Sweden) was used to measure skin perfusion and skin temperature. The probe has a fiber separation of 0.25 mm and collects perfusion data at a depth of ∼0.5 mm. The bandwidth of the system is 15 kHz. The system was calibrated before the start of the study according to the manufacturer guidelines. Perfusion values were measured at a sample rate of 33 recordings per second, and were analyzed by averaging over 1-minute intervals using Perisoft for Windows, version 2.5.5 (Perimed AB, Järfälla, Sweden).

#### Laser Speckle Contrast Imaging

A Laser Speckle Contrast imager (Pericam PSI System, Perimed AB, Järfälla, Sweden) was used to measure skin perfusion. The system uses a divergent laser beam with a wavelength of 785 nm. Perfusion images were acquired by averaging data from 42 images taken in rapid succession (acquisition time 2 seconds), over 1-minute intervals. The image size was set to correspond to a 7 cm x 7 cm area of skin and the spatial resolution of the perfusion image was 0.2 mm/pixel at a measurement distance of 25 cm. The system was calibrated according to the manufacturer recommendations. Perfusion images were further analyzed by calculating mean perfusion levels in regions of interest using PIMsoft 1.3 (Perimed AB, Järfälla, Sweden).

#### Tissue Viability Imaging

TiVi (WheelsBridge AB, Linköping, Sweden) was used to measure RBC concentration in the skin. The measurement principle has previously been described in detail [23]. The system consists of a digital camera (Canon EOS 550D) equipped with perpendicular polarization filters in front of the lens and the flash. It measures RBC concentration in arbitrary units (TiVi-index) by color analysis of the acquired images. Images were captured at 1-minute intervals, in between the LSCI measurements to avoid interference between the two techniques. Images were processed using the TiVi analysis software (TiVi version 2.1, Wheelsbridge AB, Linköping, Sweden). RBC concentration as measured by TiVi was defined as the average TiVi-index in a region of interest.

### Provocations

**Fig 1:**
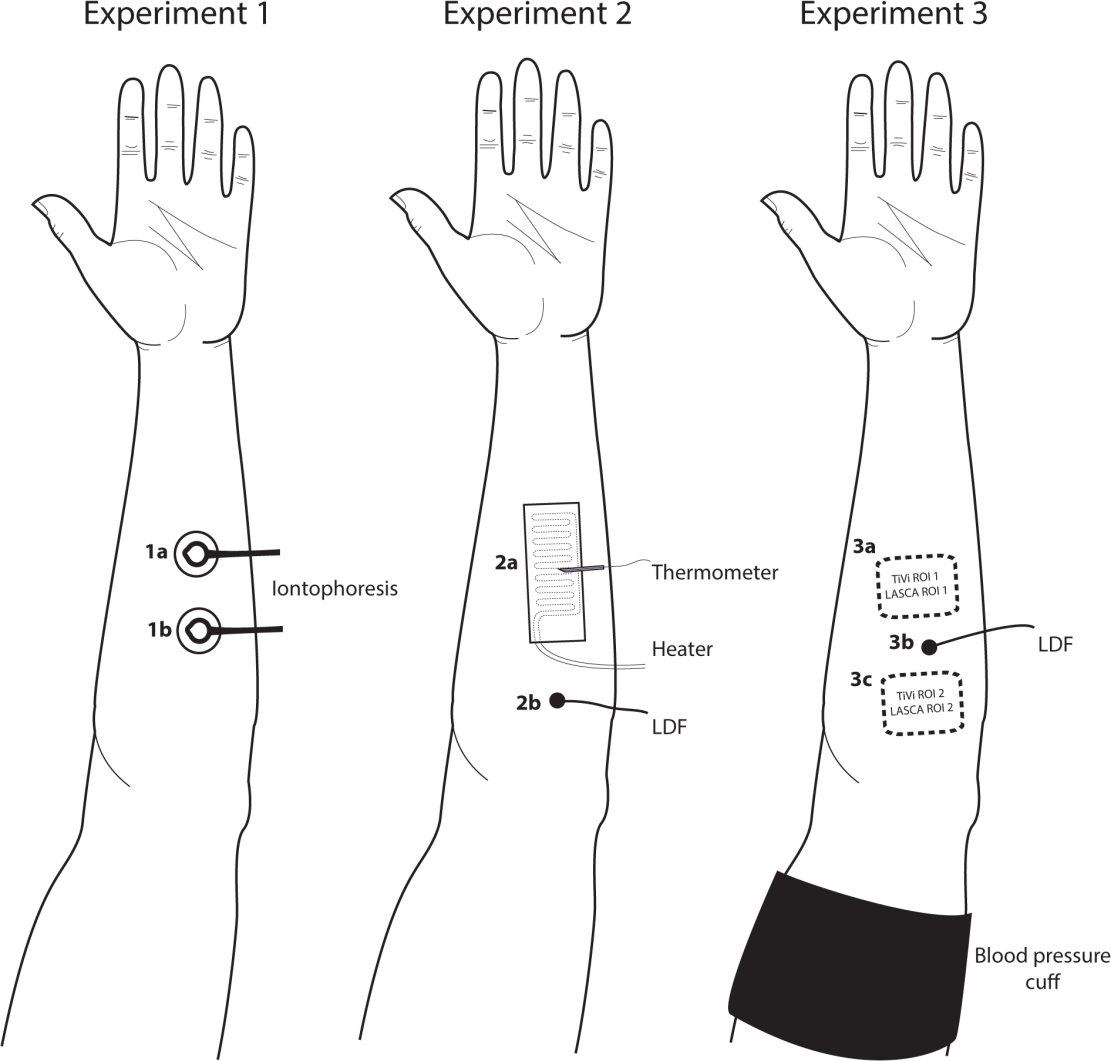
Schematic diagram of the experimental setup. The different skin sites used for measurement of microvascular responses to pharmacological and physiological provocations (see Table 2).

Three experiments with different provocations and response mechanisms were performed (Table 2 and Fig 1). Skin markings were used to ensure that the same area was analyzed for LDF, LSCI and TiVi.

#### Experiment 1—Iontophoresis of sodium nitroprusside and noradrenaline

Ten subjects (7 males) were included in the experiment; with a mean age (SD) age 24.5 ± 2.5 (range 19–29) years and mean (SD) BMI 23.7 ± 1.5 (range 22–26) kg/m^2^ were included in the experiment. Two identical battery-powered iontophoresis controllers (PeriIont 382, Perimed AB, Järfälla, Sweden) were used to deliver the drugs. Transparent, ring-shaped, drug delivery chambers with silver-silver chloride electrodes and an internal area of 1.5 cm^2^ (LI 611, Perimed AB, Järfälla, Sweden) were used to simultaneously measure with TiVi and LSCI. The distance between the silver-silver chloride electrode ring and the skin was 2 mm. Dispersive return electrodes (PF-384, Perimed AB, Järfälla, Sweden) were used to complete the electrical circuit. At the skin sites were the drug delivery electrodes were to be placed on the skin, two thermostatic laser Doppler probes were first placed to measure baseline perfusion for 2 minutes.

The drug delivery electrodes were then placed on the skin with a minimum distance of 4 cm from each other. Care was taken to avoid visible veins and irregularities in the skin. TiVi and LSCI were used to continuously measure skin microvascular responses. Both systems were positioned at a distance of 20 cm above the skin at a slightly oblique angle to avoid reflections from the electrode cover glasses. The red distance-measuring laser of the LSCI was blocked using black tape to avoid interference with the TiVi measurement. One electrode chamber was filled with SNP. The other electrode chamber was filled with NA. After a baseline measurement of 2 minutes during which measurements were made with LSCI and TiVi, NA was delivered by anodal iontophoresis for 10 minutes with a current strength of 0.02 mA, while SNP was delivered by cathodal iontophoresis for 10 minutes with a current strength of 0.02 mA, while perfusion and RBC concentration were continuously measured using LSCI and TiVi, respectively. These delivery protocols have previously been shown to minimize non-specific vasodilatory responses [2]. After the drugs had been delivered, the electrodes were removed and the laser Doppler probes were repositioned at the drug delivery sites to measure the final perfusion response to both drugs during 1 minute. For one subject, no measurement could be made with LDF because of equipment failure.

#### Experiment 2 –Local heating

Twelve subjects (10 males), mean age (SD) 25.2 ± 2.1 (range 22–29) years and mean (SD) BMI 22.8 ± 2.7 (range 19–30) kg/m^2^ were included for LDF and LSCI measurements. In a separate group of 10 subjects (6 males), mean (SD) age 26.5 ± 7.5 (range 18–38) years and mean (SD) BMI 23.2 ± 1.9 (range 20–26 kg/m^2^), an identical protocol was used in combination with TiVi measurements.

For measuring the response to local heating with LSCI and TiVi, a transparent heater was fixed to the volar side of the forearm by double-adhesive tape. The heater consisted of a 2 mm thick glass slide with a transparent heating foil (Minco SA, Aston, France) attached to its top surface. After mounting the heater to the skin, a flat thermistor was placed between the heater and the skin. The thermistor was connected to a digital thermometer to monitor the skin temperature. An LDF probe was positioned at a separate skin site, at least 5 cm from the heating glass.

A baseline was measured for 5 minutes, after which the temperature of the heating glass was set to 42°C for 40 minutes. Both systems were positioned at a distance of 20 cm above the skin at a slightly oblique angle to avoid reflections from the glass. Unfortunately, for two subjects, TiVi images could not be analyzed because of too many reflections from the glass.

#### Experiment 3 –Post-occlusive reactive hyperemia

Ten subjects (7 males), mean (SD) age 24.5 ± 2.5 (range 19–29) years and mean (SD) BMI 23.7 ± 1.5 (22–26) kg/m^2^ were included. The baseline was measured over 5 minutes with LDF, LSCI and TiVi. To obtain exsanguination, the subjects raised their arm above their head level for 1 minute. An Esmarch bandage was then tightly wrapped around the forearm, which was subsequently occluded by applying a blood pressure cuff around the upper arm with a pressure of 250 mmHg. The Esmarch bandage was then removed and the forearm was positioned at the heart level. The occlusion was maintained for 5 minutes, after which the blood pressure cuff was released. The PORH response was then recorded for 30 minutes (PORH1). Following this procedure, a new occlusion without prior exsanguination was performed for 5 minutes. Finally, the second PORH (without prior exsanguination, PORH2) was measured for 10 minutes.

To assess the site-to-site reproducibility of the image-based techniques (LSCI and TiVi), the absolute perfusion and RBC concentration during baseline were measured in two adjacent skin areas (Fig 1, sites 3a and 3c).

### Data analysis

Data was presented in terms of mean and SD. Paired Student’s t-tests were used to compare changes from the baseline values after iontophoresis in experiment 1, and to the maximum response to heating in experiment 2. In experiment 3, one-way ANOVA was used to compare the responses to different provocations for repeated measures with Holm-Sidak's multiple comparisons tests. The following responses were compared: (1) end of baseline and end of exsanguination (5 min); (2) end of PORH1 and end of occlusion (5 min); and (3) maximum response during PORH1 and PORH2 and the end of their respective prior baseline. Coefficients of variation (%CV) were calculated to evaluate inter-subject variability of the responses for the different measurement techniques and to evaluate site-to-site variability of the image-based techniques (LSCI and TiVi). The alpha level for statistical significance was set at 0.05. All statistical analyses were made with the aid of GraphPad Prism version 5.02 for Windows (GraphPad Software, San Diego California USA, www.graphpad.com).

## Results

### Experiment 1—Iontophoresis of sodium nitroprusside and noradrenaline

**Fig 2:**
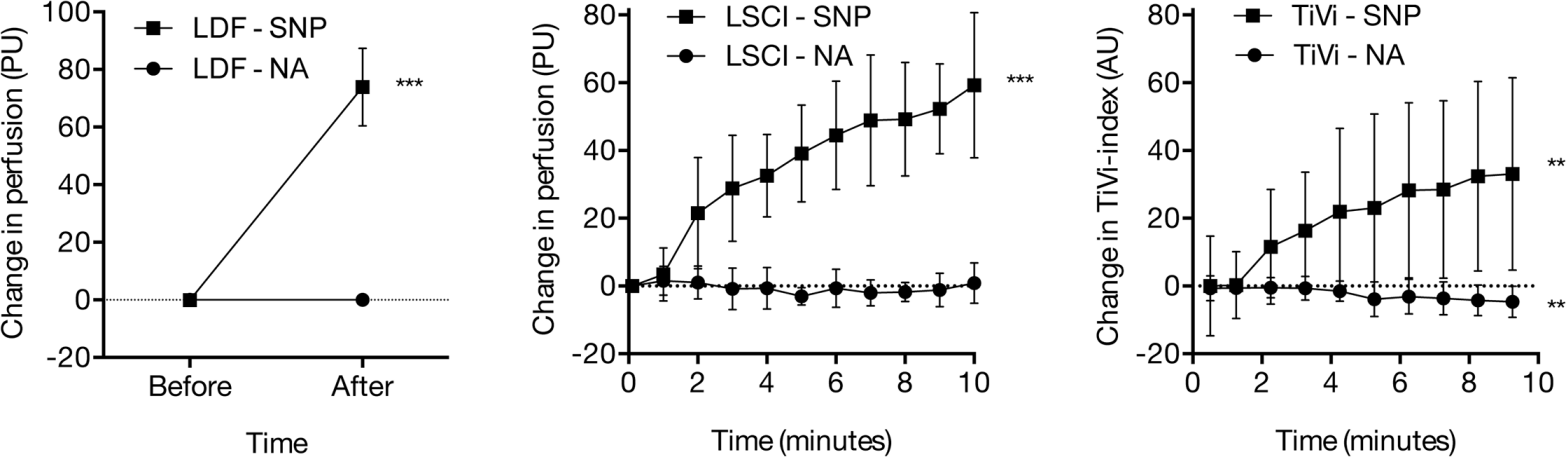
Iontophoresis of sodium nitroprusside and noradrenaline. Skin microvascular response during iontophoresis of sodium nitroprusside (SNP) and noradrenaline (NA) as measured using laser Doppler flowmetry (LDF), Laser Speckle Contrast Imaging (LSCI) and Tissue Viability Imaging (TiVi). *** indicates significant change from baseline (p < 0.001).

No change in perfusion was observed after iontophoresis of NA (LDF: p = 0.64; LSCI: p = 0.64), while RBC concentration (TiVi) decreased by 7.3 ± 8.4% (p = 0.02) (Table 3 and Fig 2). All techniques measured significant vasodilatation after iontophoresis of SNP (LDF: p < 0.001; LSCI: p < 0.001; TiVi: p = 0.01). The inter-subject variability in responses to SNP was 26.4% with LDF, 24.7% with LSCI and 27.5% with TiVi (Table 4).

**Table 3:** The mean (SD) skin microvascular response as measured using Laser Doppler Flowmetry (LDF), Laser Speckle Contrast Imaging (LSCI) and Tissue Viability Imaging (TiVi) during iontophoresis of sodium nitroprusside (SNP) and noradrenaline (NA), local heating, occlusion and post-occlusive hyperemia (PORH), with and without prior exsanguination.

Provocation		LDF (PU)	LSCI (PU)	TiVi (AU)
SNP iontophoresis	Baseline	9.4 (9.5)	40.9 (6.2)	119 (18)
	Post-provocation	82.4 (38.3)***	100 (25)***	150 (41)**
NA iontophoresis	Baseline	6.6 (2.8)	38.0 (4.4)	59.6 (11.8)
	Post-provocation	6.1 (1.6)	38.9 (5.0)	54.4 (13.3)**
Local Heating	Baseline	8.8 (3.6)	50.8 (8.0)	49.2 (32.9)
	Post-provocation	112 (55)***	151 (22)***	99.3 (28.3)***
Occlusion with prior exsanguination	Baseline	6.9 (2.6)	34.6 (5.2)	84.3 (17.0)
	Occlusion	2.4 (1.0)**	15.6 (4.7)***	69.6 (18.0)*
	PORH	51.2 (24.5) ***	93.8 (15.3) ***	137 (46) **
Occlusion without prior exsanguination	Baseline	9.0 (8.1)	34.8 (4.1)	101 (27)
	Occlusion	2.1 (0.3)	13.1 (2.6)***	101 (30)
	PORH	43.5 (30.2)**	74.0 (18.3) ***	144 (40) ***

* *p<0*.*05 vs*. *baseline*

** *p<0*.*01 vs*. *baseline*

*** *p<0*.*001 vs*. *baseline*.

**Table 4:** Inter-subject variability of skin microvascular responses as measured using Laser Doppler Flowmetry (LDF), Laser Speckle Contrast Imaging (LSCI) and Tissue Viability Imaging (TiVi) during iontophoresis of sodium nitroprusside (SNP) and noradrenaline (NA), local heating, occlusion and post-occlusive hyperemia (PORH), with and without prior exsanguination.

Provocation		LDF (PU)	LSCI (PU)	TiVi (AU)
SNP iontophoresis	Baseline	101%	15.0%	15.4%
	Post-provocation	46.4%	24.7%	27.5%
NA iontophoresis	Baseline	43.3%	11.7%	19.8%
	Post-provocation	26.0%	12.9%	24.5%
Local Heating	Baseline	40.9%	15.8%	66.9%
	Post-provocation	49.5%	14.2%	28.5%
Occlusion with prior exsanguination	Baseline	38.1%	15.2%	20.2%
	Occlusion	41.4%	30.3%	24.0%
	PORH	47,8%	16,3%	33,5%
Occlusion without prior exsanguination	Baseline	90.5%	13,6%	23.2%
	Occlusion	14,7%	27,2%	29,2%
	PORH	69,4%	24,8%	28,0%

### Experiment 2—Local heating

**Fig 3:**
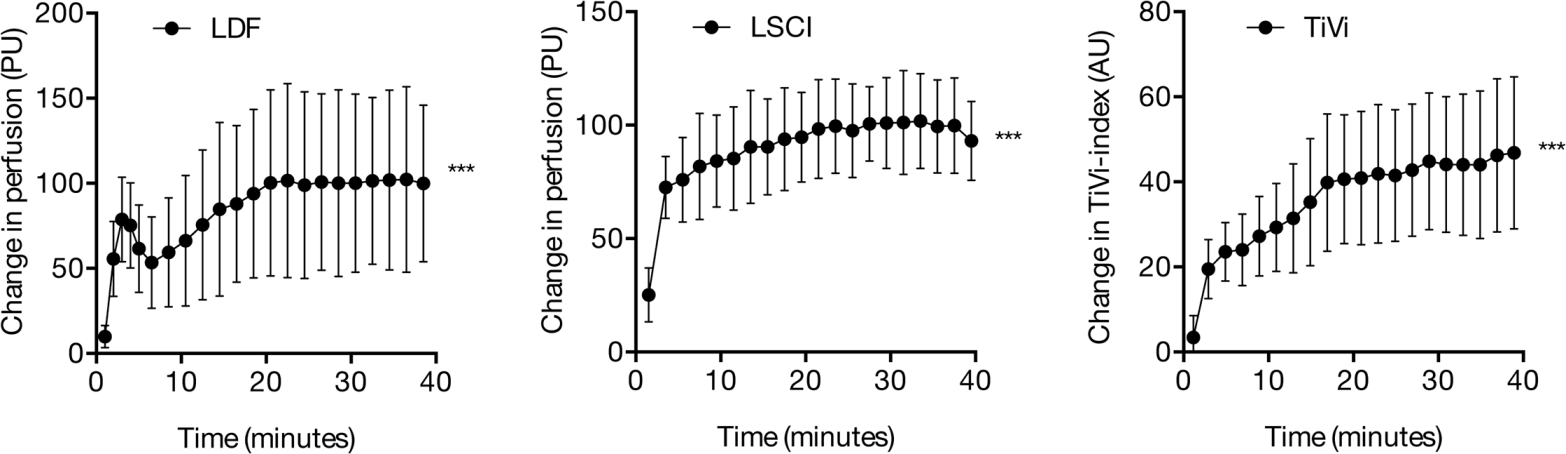
Local heating. Skin microvascular responses to 40 minutes of local heating as measured using laser Doppler flowmetry (LDF), Laser Speckle Contrast Imaging (LSCI) and Tissue Viability Imaging (TiVi). *** indicates significant change from baseline (p < 0.001).

Local heating increased skin perfusion, both as measured by LDF (p < 0.001) and as measured by LSCI (p<0.001). RBC concentration, as measured by TiVi also increased (p < 0.001) (Table 3 and Fig 3). The response during local heating was biphasic for LDF, with an initial peak after 3 minutes, followed by a slight decrease and a plateau after 20 minutes. The response with LSCI and TiVi was not biphasic but instead a rapid increase was seen during the first 2–3 minutes, followed by a more gradual increase in perfusion and RBC concentration, respectively. The LDF probe reached 42°C within 15 seconds, while the heating glass reached 42°C after 60 seconds. The inter-subject variability of the maximum response to local heating was 49.5% with LDF, 14.2% with LSCI and 28.5% with TiVi (Table 4).

### Experiment 3—Post-occlusive reactive hyperemia

**Fig 4:**
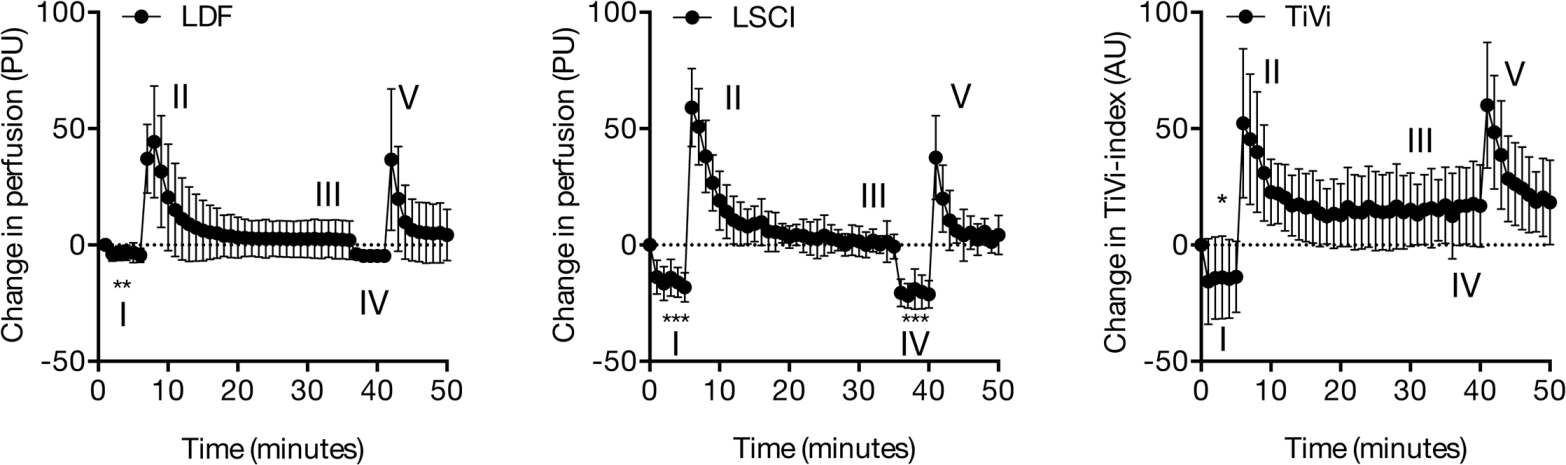
Post-occlusive reactive hyperemia. Skin microvascular responses to (I) occlusion with prior exsanguination, (II) PORH, (III) occlusion without prior exsanguination, (IV) PORH as measured using laser Doppler flowmetry (LDF), Laser Speckle Contrast Imaging (LSCI) and Tissue Viability Imaging (TiVi). * indicates significant change from baseline, p < 0.05. ** indicates p < 0.01; *** indicates p < 0.001

The mean microvascular responses to exsanguination, occlusion and post-occlusive hyperemia are shown in Table 3. LDF and LSCI detected a significant decrease in perfusion (LDF: p = 0.004; LSCI: p < 0.001) and TiVi detected a significant decrease in RBC concentration (p = 0.026) after 5 minutes of forearm exsanguination (Fig 4). Only LSCI showed a significant decrease in perfusion after 5 minutes of occlusion without prior exsanguination (p < 0.001), while a trend was seen with LDF (p = 0.053). No change in RBC concentration was observed after 5 minutes of occlusion without prior exsanguination.

With LSCI, the PORH perfusion response was significantly stronger after exsanguination compared with the PORH response after occlusion without exsanguination (p < 0.001). With LDF and TiVi, both PORH responses were similar (p = 0.43 and p = 0.18 for LDF and TiVi, respectively). The inter-subject variability after 5 minutes of occlusion with prior exsanguination was 41.4% with LDF, 30.3% with LSCI and 24.0% with TiVi (Table 4).

The site-to-site variability during baseline as assessed using within subject coefficient of variation was 2.0% for LSCI and 1.8% for TiVi.

## Discussion

The main objective of this study was to compare TiVi to LDF and LSCI in the measurement of microvascular changes in the skin of healthy subjects. The main findings are that LSCI and TiVi have different sensitivities in detecting vascular responses to pharmacological and physiological provocations in the skin. While TiVi is more sensitive than LSCI in detecting noradrenaline-induced vasoconstriction in the skin, TiVi could not detect arterial stasis after total occlusion, without prior exsanguination. These findings can be largely explained by the fact that the two techniques measure different aspects of the skin microcirculation. LSCI is similar to LDF in that it measures perfusion [22], which is related to both RBC concentration and RBC velocity. TiVi, on the other hand, is only sensitive to changes in the concentration of RBC in the skin during provocations [23].

The most commonly used technique to measure the response to vasoactive drugs delivered using iontophoresis is LDF [1]. LDF has a high temporal resolution, which makes it very suitable for monitoring the dynamics in skin perfusion, including those that occur as a result of vasomotion [25, 26]. However, LDF has a number of limitations. The technique measures perfusion in a single skin site, and spatial variations in perfusion can therefore not be directly measured without moving the probe. Also measuring vasoconstrictor response in the skin in terms of decrease in perfusion, as LDF does, is difficult [27, 28]. This is because the skin perfusion in its resting state is relatively close to the "biological zero", i.e. the mean velocity of blood cells is low in the basal state. Also, because of the lower amount of blood cells in the capillaries during vasoconstriction, the laser light tends to penetrate more into the vessels of the subdermal plexus, where the changes in perfusion may be smaller [29]. One method that has been used to facilitate the measurement of vasoconstrictor responses in the skin is to pretreat the microvascular bed with a vasodilator drug [12, 28, 30]. This method is also commonly used to normalize responses in traditional *in vitro* models [11]. Another method to predilate the microvascular bed in the skin is by local heating [6].

In agreement with previous studies in which LDF was found insensitive to measure vasoconstrictor responses, the results of this study showed that both LDF and LSCI failed to detect a decrease in perfusion during iontophoresis of NA. TiVi, which is not based on measurement of perfusion but rather on the concentration of red blood cells in the skin, has previously been found to be more sensitive than LDF in measuring vasoconstriction induced by increasing doses of NA delivered by iontophoresis [31]. The results of this study are consistent with these previous findings. TiVi was able to detect a small but consistent decrease in RBC concentration when a single dose of NA was delivered to the skin during a longer period of 10 minutes. The results of this study and previous studies therefore suggest that with TiVi as a measurement technique, responses to vasoconstricting substances can be measured in the skin without prior vasodilatory pretreatment. Although in this study, LDF and LSCI were found insensitive to drug-induced vasoconstriction, LDF has often been used successfully to measure the response to local cooling [32]. Local cooling of the skin may result in stronger vasoconstriction, and underlying mechanisms may differ from those with drug-induced vasoconstriction. This highlights the importance of using the appropriate techniques depending of the type of microvascular response studied. Alternatively, when studying vasoconstriction in the skin using LDF, local cooling may be preferred over noradrenalin iontophoresis.

The vasodilatory response to NO-donors has long been used as a control when studying endothelial function, both in *in vitro* models as well as in the skin *in vivo*, as it reflects the total vascular vasodilatory capacity, independent of endothelial function. In the skin, SNP is metabolized and produces NO, which causes relaxation of smooth muscle independent of NO-production in the endothelium. During iontophoresis of SNP in this study, all techniques could be used to detect a significant increase in perfusion and RBC concentration. With TiVi, the inter-subject variability in the response to SNP was substantially larger than with LSCI. The reason for this is not clear, but it may be related to absorption of light in the drug solution. We observed during the experiments that SNP forms an opalescent liquid layer between the skin and the iontophoresis chamber, which reduces the amount of light reflected from the underlying skin. TiVi is based on the reflection of visible light, while the laser used in our LSCI system has a wavelength beyond the spectrum of visible light (780 nm), and is possibly less affected by absorption of the drug solution in the electrode chamber during iontophoresis. Finally, it was noted in this study that the baseline RBC concentration, as measured with TiVi, varies substantially between individuals as a result of variations in skin pigmentation. Even though responses are presented as absolute changes from baseline, variations in the responses between subjects may still be in part caused by variations in skin pigmentation.

In agreement with previous studies, local heating significantly increased skin perfusion (LDF and LSCI) [33] and RBC concentration (TiVi) [34]. The microvascular response to a sudden increase in temperature is known to depend on at least two different mechanisms. It typically consists of an early peak followed by a nadir and a late plateau. The early peak is observed after three to four minutes and is mediated by local activation of cutaneous sensory nerves [35]. The late plateau is primarily mediated by the release of nitric oxide from the endothelium [15, 36], as well as sympathic noradrenergic neurotransmittors [37]. In this study, only the biphasic reaction was observed when LDF was used as a measurement technique. Since both TiVi and LSCI failed to show a biphasic response, the differences in response cannot be only due to the differences between perfusion and RBC concentration during local heating. The response differences may instead be related to the fact that the LDF probe has a diameter of 8 mm, whereas the heating glass used with the LSCI and TiVi experiments is substantially larger. This could possibly cause different temperature dynamics in the skin. In previous studies in which LDF and LSCI were used to measure the response to local heating, the dynamic perfusion response after local heating, as measured using LSCI, was only presented for single individuals [33, 38]. It could be that on a group level, the typical initial peak followed by a nadir disappears.

Exsanguination and total occlusion in the forearm are clinically used in surgery to the extremities. This is to enable operations in a bloodless field. When the occlusion is released, there is a strong vasodilatory response known as post-occlusive reactive hyperemia (PORH). Many factors have been shown to be involved in the PORH response, including sensory nerves, myogenic and metabolic factors, prostaglandins and large-conductance calcium activated potassium (BKCa) channels, as well as nitric oxide [39, 40]. The assessment of PORH in the skin has further been shown to be a sensitive indicator of atherogenesis, and recent findings suggest that the post-occlusive forearm skin reactive hyperemia response might be a valuable non-invasive method to stratify cardiovascular risk [41].

In this study, an instant decrease in perfusion was observed after exsanguination and occlusion, both with LDF and LSCI, to approximately 40% of the baseline level. The decrease in RBC concentration, as measured with TiVi, was less pronounced. This less pronounced decrease suggests that a certain amount of RBC remains in the skin. The fact that significant perfusion was measured, in spite of total occlusion, can be explained by the biological zero signal. This signal is the result of light scatter from moving particles in the tissue matrix, as well as by the residual movement of remaining RBC in the skin due to incomplete exsanguination. The second occlusion, which was done without prior exsanguination, saw a significant decrease in perfusion as measured by LSCI. On the other hand, a trend towards a significant decrease was seen using LDF. There was no change in RBC concentration as measured by TiVi, during the second occlusion. This is consistent with our previous finding [12] in which no change in RBC concentration was seen after 1 or 5 minutes of arterial occlusion. These results suggest that there is no major redistribution of blood cells from the skin into deeper vessels during 5 minutes of arterial occlusion.

The main measure of interest with most PORH provocations is the hyperemic peak, and all techniques in our experiments are able to detect this within the first minute after release of the cuff pressure. This peak is in this study presented as a rapid increase in blood flow and RBC-concentration (Table 3). The fact that TiVi is relative insensitive in measuring vasoconstriction during forearm occlusion is however important to be aware of when using the technique in PORH studies.

Single-point LDF measurements in the skin have previously been found to have a large site-to-site variability, as a result of the heterogeneity of the dermal microvasculature [42]. A few millimeters of movement of the probe has been shown to result in significant variations in measured perfusion. A lower site-to-site variability is therefore one of the main advantages of image-based measurement techniques, which are capable of measuring a microvascular response within a larger area of the skin. In our study, both LSCI and TiVi were found to have low site-to-site variabilities.

Inter-subject variability indicates the homogeneity of the response in different individuals. The method with the least inter-subject variability in this study was LSCI, with all provocations, compared to LDF and TiVi. These results are in line with a previous study comparing inter-subject variability during iontophoresis of acetylcholine, where LSCI had an inter-subject coefficient of variation of roughly half that of LDF [43]. In another study, the inter-subject variability of LDF after iontophoresis ranged from 13% to 69% [44].To the best of our knowledge, TiVi has not previously been compared with other techniques regarding inter-subject variability. The greater inter-subject variability of TiVi observed in our study should be taken to account when determining the study population size in the design of future experiments.

This study has a number of limitations. The three experiments were performed using different subject groups, where male subjects were slightly over-represented. However, for each provocation, each subject was studied using different measurement techniques. Since the purpose of the study was to compare measurement techniques, we consider it unlikely that the selection of subjects could have biased the conclusions. All subjects included in this study had light skin types (Fitzpatrick scale I or II). Since melanin is a strong absorber of visible light, the sensitivity of the measurement techniques, in particular TiVi, may be lower in subjects with darker skin types. However, the relative changes in the concentration of RBC can still be investigated by subtracting a baseline image, recorded before the start of a provocation, from all images recorded during the experiment. This is feasible because the melanin content of the skin can be considered stable in comparison with the RBC concentration following tissue provocation [23].

Further studies in subjects with more diverse skin types are needed to investigate this. We could not simultaneously measure on the exact same skin site with all three techniques, since the probe used for LDF is in contact with the skin. However, the regions of interest for LSCI and TiVi were positioned close to the LDF probe in experiment 2 and 3.

## Conclusion

The skin microcirculation is increasingly used as a model for general vascular function by studying the microvascular response to pharmacological and physiological provocations. It is therefore important to use appropriate techniques for measurement of these microvascular responses. The results of this study indicate all techniques can accurately detect the vasodilatory response to local delivery of sodium nitroprusside. The ability of TiVi to detect the microvascular changes during forearm occlusion is limited compared with LSCI, while TiVi is more sensitive than LSCI in measuring vasoconstriction in the skin after iontophoresis of noradrenaline. Both image-based techniques have a low inter-subject variability compared with LDF. We recommend that the strengths and limitations of the different techniques be taken into account when designing future studies in which microvascular function is assessed.

## Supporting Information

S1 Dataset Measurement data from LDF, LSCI and TiVi, as well as information about the subjects who participated in the study. (XLSX)
